# Acquired Thrombotic Thrombocytopenic Purpura in the Presence of a Urinary Tract Infection: A Rare Pediatric Case

**DOI:** 10.7759/cureus.50234

**Published:** 2023-12-09

**Authors:** Monica Karas, Andrew M Joseph, Omama Ahmad, Jose M Cardenas

**Affiliations:** 1 Osteopathic Medicine, Nova Southeastern University Dr. Kiran C. Patel College of Osteopathic Medicine, Davie, USA; 2 Pediatric Hematology/Oncology, University of Florida College of Medicine, Gainesville, USA; 3 Pediatric Critical Care, University of Florida College of Medicine, Gainesville, USA

**Keywords:** plasmapheresis, caplacizumab, rituximab, urinary tract infection, acquired thrombotic thrombocytopenic purpura

## Abstract

Thrombotic thrombocytopenic purpura (TTP) is a type of microangiopathic hemolytic anemia that rarely presents in the pediatric population. This life-threatening disorder manifests as severe consumptive thrombocytopenia and disseminated micro-thromboemboli, leading to organ ischemia. Here, we present a case of an acute first-time episode of acquired TTP in a 17-year-old African American female with a past medical history of obesity, recurrent urinary tract infections, and dysfunctional uterine bleeding managed with oral contraceptives. The disorder’s insidious onset was only preceded by a urinary tract infection managed as an outpatient with oral cefdinir for four days before symptoms worsened. The patient was admitted to the pediatric intensive care unit with microangiopathic hemolytic anemia, severe thrombocytopenia, low von Willebrand factor-cleaving protease (*ADAMTS13*) activity, hypofibrinogenemia, gross hematuria, and acute kidney injury. Further workup was significant for a positive urine culture for *Escherichia coli*. Her hospital course was complicated by an acute ischemic stroke. The patient’s TTP was managed by five sessions of plasmapheresis (PLEX), two once-weekly doses of rituximab, five doses of caplacizumab, three doses of high-dose solumedrol, and six days of high-dose prednisone. This regimen led to an overall uptrend in platelet counts toward normal and resolved her kidney injury. Currently, the patient continues to recover as an outpatient with no disability, managed with rituximab and caplacizumab as relapse prophylaxis. This case highlights the need for further investigation into the consideration of TTP as part of the differential diagnosis for pediatric patients presenting with severe thrombocytopenia and acute kidney injury in the absence of a significant medical history. Additionally, the utilization of rituximab, caplacizumab, steroids, and PLEX for TTP in the pediatric population should be further investigated.

## Introduction

Approximately one in a million cases per year of thrombotic thrombocytopenic purpura (TTP) is reported in children [1]. TTP is a rare and life-threatening disorder in the family of thrombotic microangiopathies (TMA), with a mortality rate of 10%-20% despite management [2]. Nearly 90% of all TTP cases are primarily present in adulthood and are twice as frequent in women compared to men [2]. However, 10% of cases are first present in children [2]. Due to its rare incidence in children, along with its increased mortality rate, rapid recognition and management of TTP are crucial to preventing strokes or other life-threatening complications [2].

Pathophysiology

TTP is characterized by vascular damage manifesting as consumptive thrombocytopenia and microangiopathic hemolytic anemia [1]. Deficiency of the specific von Willebrand factor (vWF) cleaving protease (*ADAMTS13*) has been noted to result in microthromboemboli that disseminate into the circulation, leading to organ ischemia [1]. *ADAMTS13* (A Disintegrin and Metalloprotease with ThromboSpondin Type 1 motif repeats, member 13) is a type of vWF-cleaving protease that is specifically deficient in TTP [1]. *ADAMTS13* functions by cleaving large multimers of vWF released from endothelial cells into smaller units that are not as strongly adherent to platelets [2]. Deficiency of *ADAMTS13* results in the uncontrolled release of large vWF units that bind to platelets and form microthrombi in arterial and capillary vessels [2]. These microthrombi then result in platelet consumption, which leads to tissue ischemia and microangiopathic hemolytic anemia presenting as schistocytes on peripheral blood smears [2].

Inherited vs. acquired TTP

*ADAMTS13* deficiency can be inherited through mutations in the *ADAMTS13* gene but is rare, found in 2% of all TTP cases [2]. On the other hand, acquired TTP occurs through antibodies formed against *ADAMTS13* by an autoimmune mechanism [1,2]. Predisposing factors known to increase the risk of acquired TTP include female gender, black ethnicity, and obesity [2]. Non-idiopathic TTP can present with signs of comorbid conditions in 50% of all TTP cases [2]. These conditions could have existed previously or occurred simultaneously with TTP [2]. Clinical disorders associated with TTP include bacterial infections, autoimmune diseases such as systemic lupus erythematosus and antiphospholipid syndrome, pregnancy, HIV infection, pancreatitis, cancers, and organ transplantation [2]. TTP can be induced by drugs, including mitomycin C, cyclosporine, quinine, clopidogrel, and ticlopidine [2]. The pediatric non-idiopathic TTP cases reported were found to be triggered by infections and autoimmune diseases [2].

Diagnosis

The presence of *ADAMTS13* antibodies definitively confirms TTP from other differential diagnoses such as other TMAs, hematologic malignancies, or hemolytic uremic syndrome [1,2]. *ADAMTS13* levels of less than 10% confirm the diagnosis of TTP, along with increased antibody titers against *ADAMTS13* [2]. Additionally, the PLASMIC score is used in patients with a platelet count lower than 150,000 per microliter of schistocytes on peripheral blood smear, increasing suspicion for TTP while awaiting *ADAMTS13* results, who will likely benefit from prompt plasma exchange treatment [1,2]. PLASMIC stands for the score’s seven components: platelet count, combined hemolysis variable, absence of active cancer, absence of stem cell or solid organ transplant, MCV, INR, and creatinine [3]. Low risk is consistent with a PLASMIC score less than or equal to four, indicating that other differential diagnoses should be considered [3]. Intermediate risk, associated with a PLASMIC score of five, warrants obtaining *ADAMTS13* testing and awaiting results to determine the need for plasma exchange [3]. A PLASMIC score greater than or equal to six is associated with a high risk of TTP immediate plasma exchange while awaiting *ADAMTS13* testing results is recommended [3]. 

Clinical presentation

Acute phases of TTP commonly present with severe thrombocytopenia less than 30 x 10^9^ per liter [1]. They also present with microangiopathic hemolytic anemia, characterized by schistocytes present on a peripheral blood smear and symptoms such as purpura, weakness, and dyspnea [2]. Nearly 60% of patients present with brain ischemia, which manifests as a broad range of neurological features, including altered mental status, headaches, strokes, seizures, and transient focal deficits [1]. Heart ischemia presents in 25% of patients and ranges from electrocardiographic abnormalities to the most severe myocardial infarction [2]. Isolated proteinuria and hematuria are common manifestations of renal ischemia [2]. Acute renal failure is not a common renal presentation in TTP, with serum creatinine commonly below 2 mg/dL [2]. Severe TTP, on the other hand, can present with acute renal kidney injury in 10% to 27% of patients [2]. 

Microangiopathic hemolytic anemia in TTP presents with a high reticulocyte count, low haptoglobin, and elevated lactate dehydrogenase (LDH) [2]. Schistocytes present on peripheral blood smears are the hallmark of TTP [2]. Coomb’s test is usually negative in TTP, and coagulation factors are within the normal range [2]. Renal function tests usually reveal hematuria and mild proteinuria of 1 to 2 grams per day [2]. If acute kidney injury is present, which is rare, plasma creatinine levels will be greater than 2 mg/dL [2]. Troponin I levels can also be increased (>0.25 ng/mL) despite a lack of cardiac involvement in 60% of cases [2].

In pediatrics, TTP is often misdiagnosed as hemolytic uremic syndrome, immune thrombocytopenia, Evans syndrome, or malignant hemopathy due to the common nonspecific features it shares with these diseases, such as fatigue and purpura [2]. With prompt treatment, the average survival rate from the first episode of TTP is 80%-90%. However, many TTP survivors report long-term deficits in their quality of life [2]. These include neurocognitive deficits, arterial hypertension, and major depression, which may lead to increased mortality [2].

Management

First-line therapy includes plasma exchange therapy to replenish *ADAMTS13* levels [2]. Steroids and rituximab, a humanized anti-CD20 monoclonal antibody, have been shown to target the *ADAMTS13* autoantibodies to prevent further destruction [2]. Other drugs that can be used as part of the treatment plan include pulses of cyclophosphamide, vincristine, or cyclosporine A [2]. New agents include caplacizumab, which is an anti-vWF humanized immunoglobulin fragment that prevents vWF from interacting with platelets and forming thrombi, improving consumptive thrombocytopenia and reducing the risk of micro-thromboemboli [4]. Caplacizumab was shown to quickly normalize platelet count, reduce the recurrence of TTP, and decrease the incidence of TTP-related death [4].

## Case presentation

A 17-year-old African American female with a medical history significant for obesity, dysfunctional uterine bleeding, and recurrent urinary tract infections (UTI) was transferred to our tertiary hospital for evaluation of thrombocytopenia. She was in her usual state of health until she experienced dysuria and flank pain seven days before admission. Three days later, she was seen at an outside emergency room (OER) and was prescribed cefdinir 300 milligrams (mg) twice daily for a UTI. Accidentally, she took double the amount of the prescribed dose for two days. She developed worsening fatigue and weakness with new-onset hematuria and emesis and returned to the OER. There, she was found to have a hemoglobin of 8.4 grams per deciliter (g/dL) and a platelet count of 8 x 10^9^ per liter with a negative pregnancy test. She was transferred to our tertiary hospital for further management and workup. No transfusions or interventions were completed before the transfer.

The patient had a history of irregular menses with menorrhagia managed with oral contraceptives. Her family history was significant for hypertension and type 1 diabetes; she denied any personal or family history of bleeding disorders. No previous use of anticoagulants was reported. 

During the initial encounter, the patient was afebrile and breathing at a rate of 16 breaths per minute (normal range: 12 to 18 breaths per minute). The patient was normotensive at 119/74 mmHg (normal range: 90/60 mmHg to 120/80 mmHg) with strong pulses. On a physical exam, the patient exhibited purpura on her buccal mucosa and bilateral lower extremities that had appeared a few hours before admission. The patient was also pale and appeared fatigued but was alert and oriented. 

Tertiary hospital emergency department course

At the tertiary hospital’s emergency department, complete blood count (CBC) labs were significant for low hemoglobin of 6 g/dL (normal range for females: 11.6 to 15 g/dL), low hematocrit of 17% (normal range for females: 35.5% to 44.9%), high uncorrected reticulocyte count of 6.1% (normal range 0.5% to 2.5%), high white blood cell count of 11.9 x 10^9^/L (normal range 3.4 x 10^9^/L to 9.6 x 10^9^/L), and low platelet count of 12 x 10^9^/L (normal range 150 to 450 x 10^9^/L). Further hematology labs, including undetectably low haptoglobin, a high LDH of 1,251 U/L (normal range 140 to 280 U/L), and a high total bilirubin of 2.3 mg/dL (normal range 0.1 to 1.2 mg/dL) indicated the presence of hemolytic anemia. Urinalysis on admission was negative for bacteria, leukocyte esterase, and nitrites but positive for blood. Differential diagnoses included Evans syndrome, immune thrombocytopenia purpura (ITP), thrombotic thrombocytopenic purpura (TTP), acute myelogenous leukemia (AML), hemolytic uremic syndrome (HUS), and hemophagocytic lymphohistiocytosis (HLH). She was then admitted to the pediatric hematology service for further evaluation and management of her thrombocytopenia and anemia of unclear etiology.

Pediatric hematology and oncology service course

At the tertiary hospital’s pediatric hematology service, the patient was initially placed on prednisone 60 mg twice daily for suspicions of TTP. On hospital day (HD) two of admission, the patient had decreased oral intake, palpitations, and dizziness. Bruising and petechiae were also noted on her left upper extremity and left chest. She was then transitioned to methylprednisolone, 1,000 mg daily, for increasing concerns about TTP. The patient was transfused with two units of platelets due to her low platelet count of 6 x 10^9^/L that evening. However, the patient’s platelet counts only increased from 6 x 10^9^/L to 8 x 10^9^/L after transfusion, increasing the index of suspicion for TTP. The patient was transferred to the pediatric intensive care unit (PICU) for a rapid decline in platelet count despite platelet transfusion.

Pediatric intensive care unit (PICU) course

During the PICU admission on HD 3, flow cytometry was significant for schistocytes, severe anemia, and thrombocytopenia, further supporting the diagnosis of TTP. *ADAMTS13* levels were found to be very low (9%, normal range is > 60%), and *ADAMTS13* antibodies were found to be high (43%, normal range is < 30%), definitively confirming the diagnosis of TTP. The patient remained afebrile and was transfused with one unit of packed red blood cells and one unit of platelets for the placement of a temporary hemodialysis catheter into the right internal jugular vein.

**Figure 1:**
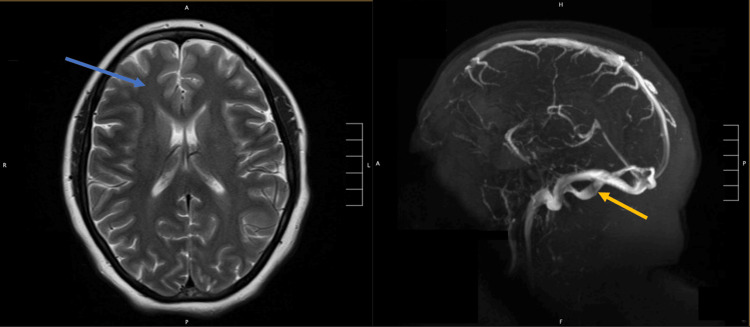
T2-Weighted MRI (left) revealed a subacute frontal infarct (blue arrow) and MRV (right) revealed a cerebellar lacunar infarct (orange arrow); both findings confirmed that the patient had a stroke. MRI: Magnetic resonance imaging; MRV: Magnetic resonance venography

On HD 3, the patient had a generalized tonic-clonic seizure lasting less than three minutes, with postictal agitation, altered mental status, and urinary incontinence, managed with a single dose of intravenous (IV) Lorazepam and dexmedetomidine infusion for sedation, and IV levetiracetam 1,000 mg twice daily for seizure prophylaxis. Head computed tomography (HCT) was unremarkable, and an electroencephalography (EEG) showed diffuse background slowing without any epileptiform discharges. The differential diagnosis for this seizure was an ischemic stroke or generalized tonic-clonic seizure induced by TTP. A T2-weighted magnetic resonance imaging (MRI) of the brain with and without contrast, in addition to a magnetic resonance venography (MRV), revealed subacute right frontal and acute cerebellar lacunar infarcts, confirming that the patient had a stroke (Figure 1). Although this may have been likely due to TTP, the multiple platelet transfusions most likely accelerated the development of the stroke by allowing for the formation of increased microthrombi.

TTP was managed with plasmapheresis (PLEX), rituximab, prednisone, and caplacizumab. PLEX was administered for five total sessions scheduled every other day. A few hours after each PLEX session, 11 mg of caplacizumab were administered. The day after the first PLEX session, 800 mg of rituximab (375 milligrams per square meter) was given. Rituximab was then administered once a week for four total weeks on non-PLEX days to ensure that the plasma exchange did not remove the drug from circulation. High-dose methylprednisolone 10 mg/kg/day was administered for the first three days before switching to prednisone 1 mg/kg/day daily until the fifth and last PLEX session on day 12 when a prednisone taper was scheduled. When all five sessions of PLEX were completed, 11 mg of caplacizumab was administered daily for 30 days since the first PLEX session. During her hospital stay, she then received five total sessions of PLEX held every other day, two doses of once-weekly 800 mg rituximab, and five doses of 11 mg caplacizumab administered after PLEX.

The patient’s infectious workup was negative for human immunodeficiency virus (HIV), SARS-CoV-2, and tuberculosis (TB). Autoimmune workup that included Sjogren’s syndrome and other inflammatory processes was also unremarkable. A repeat urine culture was positive for *Escherichia coli* (*E. coli*), initially treated with ceftriaxone and narrowed to amoxicillin for a total of 10 days.

By HD 5, the patient’s mental status continued to be altered and dependent on nasogastric feeds. By HD 6, after the second session of PLEX, her mental status had improved significantly; she was alert, active, and tolerating oral feeds. The patient’s platelets continued to trend upward to 14 x 10^9^/L from 5 x 10^9^/L the day before. Despite the fact that the patient’s neurologic status was not variable and was still not at baseline due to her stroke, she steadily improved. By HD 7, she was alert and oriented, and her platelets increased to 22. The repeat LDH was 229 U/L, much improved compared to 1,251 U/L on admission and indicated improving hemolytic anemia.

By HD 10, her neurologic status returned to baseline, with a normal platelet count of 176 x 10^9^/L. PLEX sessions were stopped, and she continued taking two more doses of rituximab once weekly and caplacizumab daily for a total 30 days after her first session of PLEX. Antiepileptic drugs were discontinued, and steroids were weaned. Her hemolytic anemia resolved on HD 14 with a normal LDH level. The patient was then discharged with a full return to neurologic baseline and no disability. 

**Figure 2:**
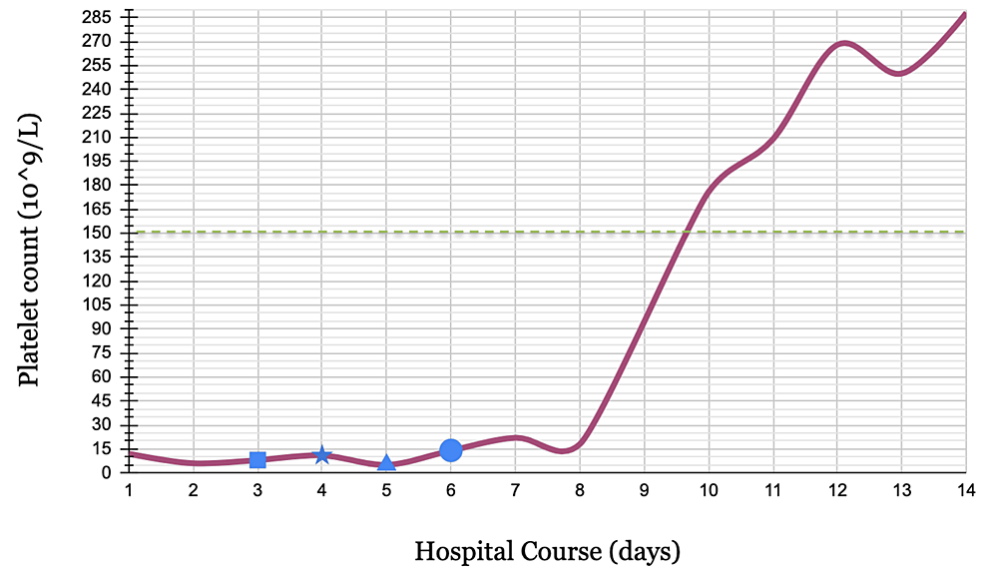
Trends of platelet count throughout the hospital course Square: start of solumedrol course; Star: start of PLEX sessions and caplacizumab; Triangle: start of rituximab; Circle: start of prednisone course and stop of solumedrol; Green dashed line: low normal reference range of platelet count (150 x 10^9^/L)

The patient’s overall platelet counts throughout her hospital course and after five sessions of PLEX, two doses of rituximab, and five doses of caplacizumab are shown in Figure 2.

Acute kidney injury

**Figure 3:**
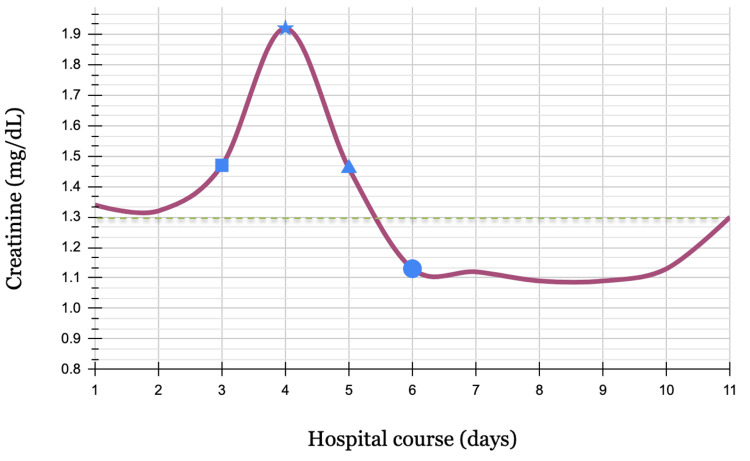
Trends in serum creatinine throughout the hospital course Square: start of solumedrol course; star: start of PLEX sessions and caplacizumab; triangle: start of rituximab; circle: start of prednisone course and stop of solumedrol; green dashed line: high normal of creatinine (0.84 mg/dL)

The patient’s increased creatinine is likely due to acute kidney injury and is secondary to TTP. A renal ultrasound was unremarkable. Although rare, acute kidney injury can be attributed to TTP due to the microthrombi resulting in kidney ischemia. The patient’s estimated glomerular filtration rate (eGFR) initially was 53 on admission, which then increased to 72 and stabilized since day six of admission. Shown in Figure 3 below is the creatinine level trend throughout the hospital course.

## Discussion

Pediatric cases of thrombotic thrombocytopenic purpura are very rare yet life-threatening if not managed properly [2]. TTP in adults commonly presents with the pentad of thrombocytopenia, fever, hemolytic anemia, renal injury, and neurologic abnormalities [2]. Although our patient only met 4 out of the 5 criteria, her abnormally low platelet count of 8 x 10^9^ per liter on admission, along with a high-risk PLASMIC score of 7, was high risk for severe *ADAMTS13* deficiency, as shown in Table 1.

**Table 1:** PLASMIC score components and scoring guide MCV: mean corpuscular volume; INR: international normalized ratio; mg/dL: milligrams per deciliter; fL: femtoliters; mmol/L: millimoles per liter

PLASMIC score components	Patient’s values on admission	Score
Platelet count < 30 x 10^9^/L	12 x 10^9^ /L	L+1
Hemolysis: Reticulocyte count > 2.5% Haptoglobin undetectable Or Indirect bilirubin >2.0 mg/dL (34.2 micromol/L)	Microangiographic hemolytic anemia: Reticulocyte count uncorrected = 6.1% Haptoglobin = undetectable Indirect bilirubin = 2.3 mg/dL	+1
Active cancer, or treated for cancer within the past year	No	+1
History of solid - organ or stem-cell transplant	No	+1
MCV < 9.0 x 10^-14^L (<90 fL)	MCV = 74.8 fL	+1
INR < 1.5	INR = 1.2	+1
Creatinine < 2.0 mg/dL (176.8 mmol/L)	Creatinine = 1.34 mg/dL	+1
	Total Score	7

Our patient had a medical history of recurrent UTIs and initially presented with UTI symptoms, including burning during urination, which were confirmed by a urine culture at the outside emergency room and treated with oral cefdinir. On day four of cefdinir treatment, she experienced worsening fatigue, weakness, tachypnea, dizziness, new-onset easy bruising, and gross hematuria, which prompted another visit to the OED and a subsequent transfer to admission at the tertiary hospital for further evaluation.

Since our patient did not have a family history of TTP and considering its late onset, hereditary TTP is ruled out in this patient. She received cefdinir for recurrent UTI multiple times in the past without any adverse effects such as easy bruising or gross hematuria and does report menorrhagia and irregular menses regulated by oral contraceptives but denies any diagnoses of anemia or low hemoglobin in the past, ruling out a hemoglobinopathy or coagulation disorders. Since the direct antiglobulin test was negative, Evans syndrome was ruled out. Also, the infectious workup was negative for HIV, TB, cytomegalovirus, Epstein-Barr virus, and parvovirus. Additionally, the respiratory viral panel was negative, ruling out viral infections. The immunology workup was also unremarkable and included CD20 (+) B cells, CD3 (+) T cells, and immunoglobulin A (IgA), ruling out immune disorders such as selective IgA deficiency or leukemia. Lastly, autoimmune workup was also negative for double-stranded DNA antibodies, islet cell antibodies (IA2 autoantibodies), anti-systemic sclerosing A and B antibodies (anti-SSA/anti-SSB), Zinc T8 autoantibody, and antinuclear antibody. The unremarkable autoimmune workup, therefore, rules out any autoimmune disorders such as systemic lupus erythematosus or Sjogren’s syndrome.

Since the urine culture on HD 3 was positive for *E. coli* despite four days of cefdinir, the UTI was considered not resolved when symptoms worsened. Therefore, the patient’s acute UTI that was not resolved by a four-day course of cefdinir may have likely induced an acute episode of acquired TTP. Another differential diagnosis is idiopathic TTP, which may be likely or unlikely due to the patient’s prior UTIs that did not present with worsening symptoms. However, the presence of UTI symptoms that persisted into HD 3 with a positive urine culture and despite four days of cefdinir indicates that the UTI could be the culprit in this patient.

Mechanisms of UTI-induced acquired TTP have been discussed in the literature, but such cases are very rarely reported clinically, especially in pediatrics [5]. A possible mechanism of UTI-induced acquired TTP is that antibodies being produced against the bacterium causing the UTI resulted in molecular mimicry and targeted *ADAMTS13* as well [5]. Considering this patient’s recurrent UTIs, her immune system might have formed a hyperactive response that produced antibodies that target but are not specific for *E. coli* and were able to target *ADAMTS13* as well through molecular mimicry [5]. Another possible mechanism is that the infectious state due to the chronic recurrent UTIs resulted in higher levels of vWF antigen and inflammatory markers interleukin-8 and tumor necrosis factor alpha, leading to the formation of large vWF multimers that then consumed platelets to form microthrombi [5]. Interleukin-6 normally cleaves those large vWF multimers to prevent the formation of microthrombi in the flowing vasculature, but the recurrent UTIs precipitate an infectious state where interleukin-6 does not function in static conditions such as a UTI [5]. The microthrombi can then lead to consumptive thrombocytopenia and, therefore, an acquired immune deficiency of *ADAMTS13* [5].

Management of pediatric thrombotic thrombocytopenic purpura 

Management of TTP revolves around removing *ADAMTS13* autoantibodies from plasma, preventing antibodies from recurring, and breaking down microthrombi that will or may have formed to prevent embolism and stroke [2]. Plasmapheresis, rituximab, steroids, and, most recently, caplacizumab are commonly used to treat TTP in an inpatient setting [2].

PLEX is one of the first-line treatments for TTP [2]. PLEX removes *ADAMTS13* antibodies from plasma and replaces them with healthy plasma that contains replenished levels of *ADAMTS13* and platelets [2]. The plasma replacement regimen is initially 1.5 times the patient’s plasma volume, followed by one times the patient’s plasma volume. Some studies recommend that PLEX should be started as soon as the patient is diagnosed and conducted daily until symptoms of multi-organ ischemia have resolved, platelets have recovered and are stable, and hemolysis is corrected [2]. In addition to PLEX, steroids can help reduce the immune system’s inflammatory response to prevent further production of *ADAMTS13* autoantibodies [2]. High-dose methylprednisolone at 10 milligrams per kilogram per day (mg/kg/day) for three days, followed by 2.5 mg/kg/day, was found to be effective as an adjunctive treatment to PLEX in newly diagnosed TTP patients [2]. Current literature recommends daily PLEX for 30 days [2]. However, this also indicates that any medication given to the patient is cleared out of the patient’s system every day. To provide a chance for the rest of the medications to exercise their effects, PLEX was timed every other day until the patient recovered and returned to baseline after a total of five PLEX sessions.

Rituximab is another treatment option in addition to PLEX. Rituximab is a humanized anti-CD20 monoclonal antibody that depletes B cells to suppress *ADAMTS13* autoantibody production [6]. It was shown to be effective in PLEX remission after four weekly doses of 375 milligrams per meter squared per dose [2]. In four retrospective studies, remission was achieved in 89% of cases of TTP that did not optimally respond to standard treatment of PLEX [2]. In two prospective studies of 47 patients with recurrent TTP, 98% of patients achieved remission within the first month of diagnosis, and no relapse occurred after one year of follow-up [2]. The half-life of rituximab ranges from 76.3 to 205.8 hours, and clinical studies have shown that once-weekly doses for a maximum of four doses had decreased side effects when compared with eight doses [2,6]. These results indicate that rituximab is effective when used as an adjunct to PLEX but should be administered once weekly for a maximum of four doses after a PLEX session is conducted to prevent breakdown [2,6]. Therefore, rituximab was administered once weekly, timed after a PLEX session, in this patient for a total of two doses.

Finally, caplacizumab is a newly approved drug that has shown benefit, especially in patients with acquired TTP [4]. Caplacizumab is an anti-vWF humanized immunoglobulin fragment that prevents vWF from interacting with platelets and forming thrombi, improving consumptive thrombocytopenia and reducing the risk of micro-thromboemboli [4]. In the HERCULES study, a double-blind, multicenter, phase-three, randomized controlled trial that consisted of 145 patients, caplacizumab was administered during daily PLEX for 30 days [4]. Caplacizumab was shown to quickly normalize platelet count, reduce the recurrence of TTP, and decrease the incidence of TTP-related death [4]. Administered alone, caplacizumab was shown to have a high rate of relapse after stopping the drug 30 days after the last PLEX session [4]. Caplacizumab first received Food and Drug Administration (FDA) approval for use in adults in February 2019 [4]. It did not receive FDA approval for use in pediatrics since no pediatrically acquired TTP patients were enrolled in the clinical trials [4]. Research studies were then published that developed pediatric dosing regimens using model-based simulations, which recommended 10 mg in children weighing greater than or equal to 40 kilograms and 5 mg otherwise [4].The half-life of caplacizumab ranges from 16 to 27 hours [4]. Since this patient receives PLEX sessions every other day, it is reasonable to deduce that caplacizumab was cleared from the patient’s system when the next PLEX session is due. Therefore, caplacizumab was administered after every PLEX session for a total of five doses.

In this patient, all four modalities mentioned above were used adjunctly to treat the patient’s acquired TTP through the following regimen: five PLEX sessions spaced every other day to normalize her platelet count, two doses of rituximab, five doses of caplacizumab, three days of high-dose methylprednisolone, and nine days of prednisone. Combining all four modalities together targets the synthesis and effects of *ADAMTS13* autoantibodies through different mechanisms to prevent relapse. PLEX removes the *ADAMTS13* autoantibodies from the plasma and replaces them with healthy *ADAMTS13*, while caplacizumab prevents clot formation to prevent further organ dysfunction from the micro-thromboemboli. Steroids help reduce the immune system’s inflammatory response at large, targeting the synthesis of these autoantibodies differently than rituximab, which specifically depletes B cells. By targeting the various facets of the immune response and the formation of *ADAMTS13* autoantibodies simultaneously instead of individually, this regimen effectively and quickly treats TTP.

In summary, this patient required a total of only five PLEX sessions, rituximab, caplacizumab, and steroids. There are no studies currently published that have combined all four treatments into one regimen. Therefore, this patient’s treatment regimen is the first reported pediatric case in the literature that experienced quick improvement in platelet count, hemolytic anemia, and acute renal kidney injury. 

## Conclusions

In conclusion, this report presents a rare pediatric case of a 17-year-old African American obese female with a medical history of menorrhagia on oral contraceptives admitted for acquired TTP in the setting of a urinary tract infection complicated by acute kidney injury, stroke, hyperglycemia, and secondary hypertension. The patient’s acquired TTP was confirmed by the low activity of *ADAMTS13* and the presence of autoantibodies to the vWF cleavage factor *ADAMTS13*. The patient was managed by five sessions of PLEX, rituximab, steroids, and caplacizumab. Further investigation should be pursued to assist clinicians in identifying acquired TTP due to its high mortality rate if not immediately treated and to study the combined multimodal therapy of PLEX, rituximab, caplacizumab, and steroids in acquired TTP. Therefore, acquired TTP should be high on the differential diagnosis if a pediatric patient with no past medical history of bleeding disorders presents with consumptive thrombocytopenia, microangiopathic hemolytic anemia, acute kidney injury, and neurologic symptoms due to its high mortality if not treated immediately, starting with plasmapheresis.
